# The Membrane-Water Partition Coefficients of Antifungal, but Not Antibacterial, Membrane-Active Compounds Are Similar

**DOI:** 10.3389/fmicb.2021.756408

**Published:** 2021-11-05

**Authors:** Pavel E. Volynsky, Alexandra I. Smirnova, Sergey A. Akimov, Svyatoslav S. Sokolov, Fedor F. Severin

**Affiliations:** ^1^Laboratory of Biomolecular Modeling, Shemyakin-Ovchinnikov Institute of Bioorganic Chemistry, Russian Academy of Sciences (RAS), Moscow, Russia; ^2^Department of Molecular Energetics of Microorganisms, Belozersky Institute of Physico-Chemical Biology, Lomonosov Moscow State University, Moscow, Russia; ^3^Faculty of Bioengineering and Bioinformatics, Lomonosov Moscow State University, Moscow, Russia; ^4^Laboratory of Bioelectrochemistry, A.N. Frumkin Institute of Physical Chemistry and Electrochemistry, Russian Academy of Sciences (RAS), Moscow, Russia

**Keywords:** sterol, membrane, pore, antibacterial, antifungal

## Introduction

Sterols are essential components of eukaryotic membranes and play a structural role, increasing the resistance of the phospholipid bilayer to various stresses (Evans and Rawicz, 1990; Zhelev and Needham, 1993; Michalak et al., 2013; Hannesschlaeger et al., 2019). Concentrations of sterols are especially high in the plasma membrane, typically comprising 30–40% of the total lipid content (Ejsing et al., 2009; Subczynski et al., 2017). Most prokaryotes lack sterol biosynthesis machinery and thus their plasma membranes are sterol-free. Many antimicrobial drugs disrupt the plasma membrane phospholipid bilayer. Here we ask whether antifungal and antibacterial compounds are fundamentally different in their physico-chemical properties which reflect their interactions with plasma membranes with distinct lipid compositions. A drug-membrane interaction can be divided into two stages: first, a drug moves from the aqueous phase into the water-membrane interface saturated by polar and charged groups. Next, it penetrates into the hydrophobic core of the phospholipid bilayer. Thus, we estimated the energy costs of these two steps for the antibacterial and antifungal compounds commonly used for medical and/or agricultural purposes. We have deliberately limited our analysis to such practically used compounds because their biological activities are, obviously, characterized more thoroughly than the ones of the chemicals used, for instance, for research purposes only. It appeared that while antibacterial compounds differed considerably in terms of the energy costs, antifungal compounds displayed a significant degree of similarity. This finding suggests a common mechanism for the interaction between the antifungal membrane-active compounds and the fungal plasma membrane.

### Sterols Stabilize Membranes by Preventing Pore Formation

**Figure 1:**
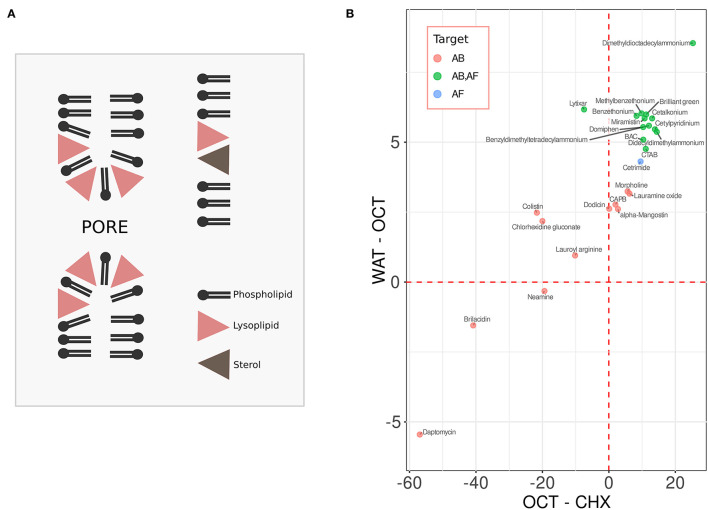
**(A)** sterols protect membrane against pore formation caused by lysolipids and similar compounds. **(B)** the water-octanol (WAT-OCT) and the octanol-cyclohexane (OCT-CHX) change in energy of the compounds with antibacterial (AB, red), antifungal (AF, blue), and the dual activity (AB, AF, green). See text for details.

While bacteria do not possess sterol biosynthesis machinery and thus are typically devoid of sterols, in eukaryotes sterols may reach up to 50 mol.% of total lipids in the plasma membrane (Mouritsen and Zuckermann, 2004; Ejsing et al., 2009; Subczynski et al., 2017). Sterols are especially efficient in increasing the resistance of lipid bilayers with respect to lysis via pore formation caused by the membrane-active chemicals (Sot et al., 2014; Mattei et al., 2015; Caritá et al., 2017). The mechanism of this protection can be illustrated using the example of lyso-forms of phosphocholine lipids. These compounds are similar to regular phosphocholine lipids but lack one of two hydrophobic tails of the molecule. Thus, while the molecules of the regular phosphocholine lipids are of cylindrical or slightly conical shape, the shape of the lysolipid derivatives is inverse conical with the wider polar side positioned close to the membrane-water interface (Fuller and Rand, 2001). Such an arrangement leads to the alteration of the lipid monolayer curvature favoring pore formation (see Figure 1A). The effective shape of sterol molecules is also non-cylindrical—they are rather conical, and the base of the cone is typically embedded into the hydrophobic core (Fuller and Rand, 2001; Kollmitzer et al., 2013). In this way sterols can compensate for the curvature alterations caused by the lysolipids, preventing pore formation and irreversible membrane rupture (Figure 1A) (Karpunin et al., 2005; Strandberg et al., 2012). Most membrane-active compounds, e.g., amphipathic antimicrobial peptides, disrupt membranes in a manner similar to lysolipids: in relatively high concentrations they act as a classic surfactant leading to micellization of the membrane; in lower concentrations pores are readily formed in the membrane (Tamba et al., 2010; Henderson et al., 2016; Pérez-Peinado et al., 2018). This makes sterols rather universal compounds in terms of preventing pore formation in eukaryotic membranes.

As prokaryotic membranes, unlike fungal ones, typically lack sterols, we reasoned that the membrane-active compounds targeting these groups are likely to be fundamentally different.

### Antifungal Membrane-Active Compounds Display Higher Similarity in Physico-Chemical Properties Than Antibacterial Compounds

To test this hypothesis, we first compiled a list of antifungal, antibacterial or the dual activity compounds targeting the outer membrane, which are used in medicine and/or agriculture (Figure 1B). Most of them are listed in four comprehensive reviews (Fait et al., 2019; Falk, 2019; Zhang and Ma, 2019; Anestopoulos et al., 2020). An additional literature search for such compounds added Brilliant Green and Chlorhexidine gluconate (general antimicrobial antiseptics) to our list. Also, Lytixar and Brilacidin were included according to Zhang et al. (2018), and Morpholine, 4-dodecyl-, 4-oxide were added based on (Šubík et al., 2021). Membrane-active compounds which display additional activities, e.g., oxolinic acid, which is also a DNA-gyrase inhibitor, were not included in the list. Next, we tried to characterize the basic features of their interaction with phospholipid bilayers.

A first step of an interaction between a chemical compound and a phospholipid bilayer is a transition between the aqueous phase to the aqueous-hydrophobic interface. Second, the chemical enters the hydrophobic phase of the membrane. Thus, to characterize the interaction we calculated the free energy changes for both of the transitions (Figure 1B). The energies of interaction of each chemical with the media were calculated as the sums of accessible surface areas of the atoms, weighted by the atomic solvation parameter (ASP) for different solvents. ASPs have been calculated based on the distribution of amino acids between different liquids. This approach has been successfully tested in the study of interactions of proteins with membranes (Efremov et al., 1999). To mimic aqueous and inner membrane media we have used ASP sets for water and cyclohexane, respectively. To estimate the energy on the water-membrane interface we used an ASP set for octanol, which to some extent emulates this medium (Allen, 2007). Next, we plotted these values: Y-axis shows the water-octanol (WAT-OCT) change in energy, X-axis shows the octanol-cyclohexane (OCT-CHX) differences (Figure 1B). The values corresponding to the compounds with the antibacterial activity (AB) only are shown in red, the ones corresponding to the chemicals with antifungal (AF) activity are shown in blue, the ones with the dual activity (AB, AF) are shown in green. By looking at the graph one can easily notice that the antifungal compounds display a much higher degree of clustering than the antibacterial ones. One of the antifungal compounds outside the cluster (green dot on the left hand side, Figure 1B) corresponds to Lytixar, the activity of which depends on sphingolipids (Bojsen et al., 2013). The mechanism of action of the other outlier, Dimethyldioctadecylammonium bromide (the right hand side of the graph), is also a rather special one. It does not simply disrupt the fungal plasma membrane, but makes its antigens more accessible to the immune system (De Serrano and Burkhart, 2017).

## Discussion

The clustering of the compounds with antifungal activity obviously suggests a common mechanism of their action. Analysis of the literature suggests that simple lysis of cells is not such a mechanism. Rather than puncturing the plasma membrane, these compounds seem to induce intracellular changes: inhibit the hyphal growth and/or biofilm formation, cause ROS generation, etc. (reviewed Fait et al., 2019; Falk, 2019; Zhang and Ma, 2019; Anestopoulos et al., 2020). This is consistent with the protective role of ergosterol against pore formation (Figure 1A). As the clustered compounds contain positively charged quaternary amine groups within their structures, one may suggest that they can bind to anionic xenobiotics and thus facilitate their penetration through the plasma membrane by neutralizing the negative charge. Importantly, a number of fatty acids do possess antifungal activity. The list includes palmitic, lauric, arachidonic and the other major fatty acids present in animals and plants. Among other activities, they inhibit the biosynthesis of ergosterol and induce ROS generation in fungal cells. One can easily imagine that, for instance, the benzalkonium cation binds to palmitic acid and this facilitates its transport across the phospholipid bilayer—a phenomenon which has been already shown by us for a quaternary phosphonium-based compound (Severin et al., 2010). Regardless of whether this explanation is correct or not, the clustering illustrated by Figure 1B suggests a novel approach for identifying novel antifungals. For instance, one can consider *in silico* screening of FDA-approved chemicals with physico-chemical properties similar to those of the compounds within the antifungal cluster. The data provided by this paper suggest that each such chemical may be a potential candidate for re-profiling as an antifungal.

## Author Contributions

FS and SS drafted the manuscript. PV, AS, and SA participated in the initial observations and discussion. All authors were involved in literature search and editing of the manuscript.

## Funding

This work was supported by the Russian Science Foundation (grant No. 18-14-00151).

## Conflict of Interest

The authors declare that the research was conducted in the absence of any commercial or financial relationships that could be construed as a potential conflict of interest.

## Publisher's Note

All claims expressed in this article are solely those of the authors and do not necessarily represent those of their affiliated organizations, or those of the publisher, the editors and the reviewers. Any product that may be evaluated in this article, or claim that may be made by its manufacturer, is not guaranteed or endorsed by the publisher.

## Abbreviations

ASP: Atomic Solvation Parameter
AB: Antibacterial
AF: Antifungal.
